# Quantitative Histological Validation of Diffusion Tensor MRI with Two-Photon Microscopy of Cleared Mouse Brain

**DOI:** 10.2463/mrms.bc.2015-0148

**Published:** 2016-03-30

**Authors:** Koji KAMAGATA, Aurelien KEREVER, Suguru YOKOSAWA, Yosuke OTAKE, Hisaaki OCHI, Masaaki HORI, Kouhei KAMIYA, Kouhei TSURUTA, Kazuhiko TAGAWA, Hitoshi OKAZAWA, Shigeki AOKI, Eri ARIKAWA-HIRASAWA

**Affiliations:** 1 Department of Radiology, Juntendo University Graduate School of Medicine, 2-1-1 Hongo, Bunkyo-ku, Tokyo 113-8421, Japan; 2 Research Institute for Diseases of Old Age, Juntendo University Graduate School of Medicine; 3 Research & Development Group, Hitachi Ltd.; 4 Department of Radiology, University of Tokyo; 5 Department of Radiological Sciences, Graduate School of Human Health Sciences; 6 Department of Neuropathology, Tokyo Medical and Dental University

**Keywords:** cleared brain, CUBIC, diffusion tensor imaging, mouse

## Introduction

Diffusion tensor imaging (DTI) is unrivalled in its ability to quantify nerve fiber pathways and microstructures in the central nervous system *in vivo*.^1^ However, its pathological underpinnings are not as solid as its widespread clinical application would suggest.^2^ A number of studies have already been performed to verify the accuracy of diffusion tensor parameters, comparing them with indices such as cell counts,^3^ cell size,^4^ and staining intensity^5^ in slices of postmortem tissue. Magnetic resonance imaging (MRI), however, provides three-dimensional (3D) voxel-based data, and information from two-dimensional tissue slices cannot be used for accurate verification. The tissue is also damaged by slicing, which causes microstructural changes in, for example, neurite density. We have adopted brain clearing to overcome these limitations. The brain-clearing method we use not only makes the brain optically transparent, but also enables the preparation of 3D neural network images with single-cell resolution.^6,7^ Brain clearing thus makes it feasible to investigate 3D structures inside the brain in greater detail than with standard glass-slide immunostaining, and it is expected to become a valuable technique in the field of neuroscience. Our objective here was to use 3D information gleaned from brain clearing and two-photon microscopy to verify the diffusion tensor estimates calculated from DTI data.

## Materials and Methods

### Sample preparation

One thy-1 yellow fluorescent protein [B6.Cg-Tg (Thy1-cre/ERT2,-EYFP) AGfng/J; obtained from Jackson Laboratory, Bar Harbor, ME, USA] 26-week-old male mouse was deeply anesthetized and then perfused with 25 ml of an ice-cold phosphate-buffered saline (PBS)-heparin (10 U/ml) solution followed by 25 ml of an ice-cold paraformaldehyde (PFA, 4%) solution. The brains were carefully dissected and post-fixed in 4% PFA overnight and embedded in 1% agarose for MRI. After scanning MRI, brain sections (thickness, 2 mm) were prepared by using a mouse brain matrix (Muromachi Kikai, Tokyo). Slices were then cleared by using the clear, unobstructed brain imaging cocktails and computational analysis (CUBIC) method. Briefly, 2 mm brain slice were incubated for 3 days in cubic 1, washed in PBS overnight, and then incubated in cubic 2 for 3 days. All animal protocols were approved by the Animal Care and Use Committee of Juntendo University.

### MRI

Whole brain MR images were acquired within 2 weeks from perfusion with a 7-T animal MRI system (Agilent Technologies Inc., Palo Alto, CA, USA). Four turn solenoid coil was used to acquire the images. Its inner diameter and length were 17 and 20 mm, respectively. The DTI sequence used was a 3D diffusion-weighted fast spin echo sequence with the following parameters: repetition time = 300 ms; echo train length = 4; echo time = 31.86 ms; two averages; field of view, 19.2 × 19.2 × 19.2 mm; and matrix size, 128 × 128 × 128. This yielded an image with 150-μm isotropic voxels. Two initial b = 0 s/mm^2^ images and 30 b-value (b = 1000 s/mm^2^) in 30 different directions were acquired corresponding to the Jones 30 scheme. The total imaging time was 21 h 10 min. Maps of fractional anisotropy (FA) and mean diffusivity (MD) were computed by using dTV II and VOLUMEONE 1.72, developed by Masutani et al.^8^

### Two-photon microscopy acquisition

Image acquisition was performed with a Carl Zeiss LSM 780 two-photon microscope (Carl Zeiss, Oberkochen, Germany; two-photon Chameleon laser; wavelength, 920 nm) equipped with a ×10 Plan Apochromat objective (numerical aperture, 0.45; working distance, 2 mm). Acquired images were 16-bit tiff with the following voxel dimensions (μm): x, 1.66; y, 1.66; z, 4.

### Region of interest (ROI) analysis

**Fig. 1.**
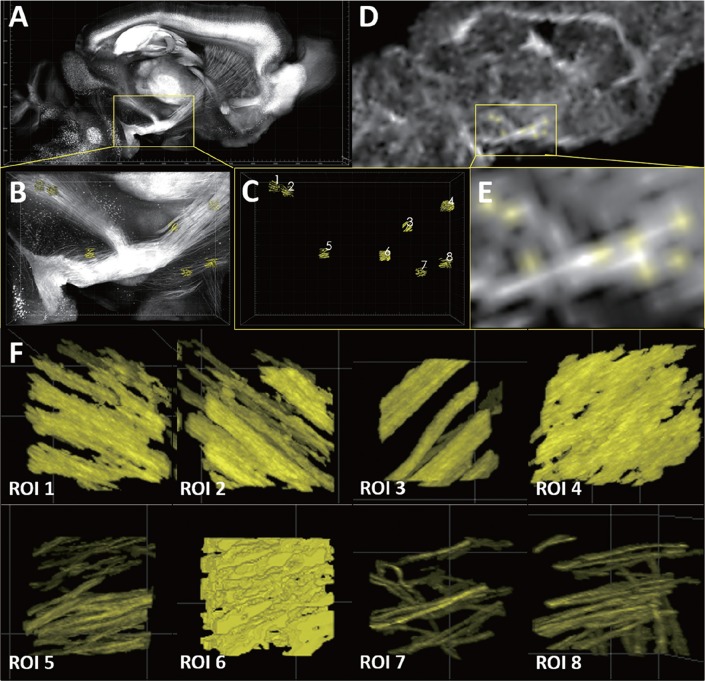
Two-photon imagings of cleared Thy-1 yellow fluorescent protein mouse brain (**A**, **B**, **C**, **F**), and a fractional anisotropy (FA) map of the same mouse brain (**D**, **E**) (medial lemniscus). (**A**) Sagittal image 1 mm to the left of the midline, acquired by two-photon microscopy. (**B**) Magnification of (**A**) centered on the medial lemniscus. The ROIs (yellow) were set randomly within the medial lemniscus. (**C**) Neurites within the ROIs are shown in yellow. (**D**) An FA map of the same cross-section as (**A**). (**E**) Magnification of (**D**). ROIs were set in the same regions as those shown in (**B**). (**F**) Histological details in selected ROIs. In all the ROIs, the fiber orientations were comparatively well aligned. FA, fractional anisotropy; ROI, region of interest.

**Fig. 2.**
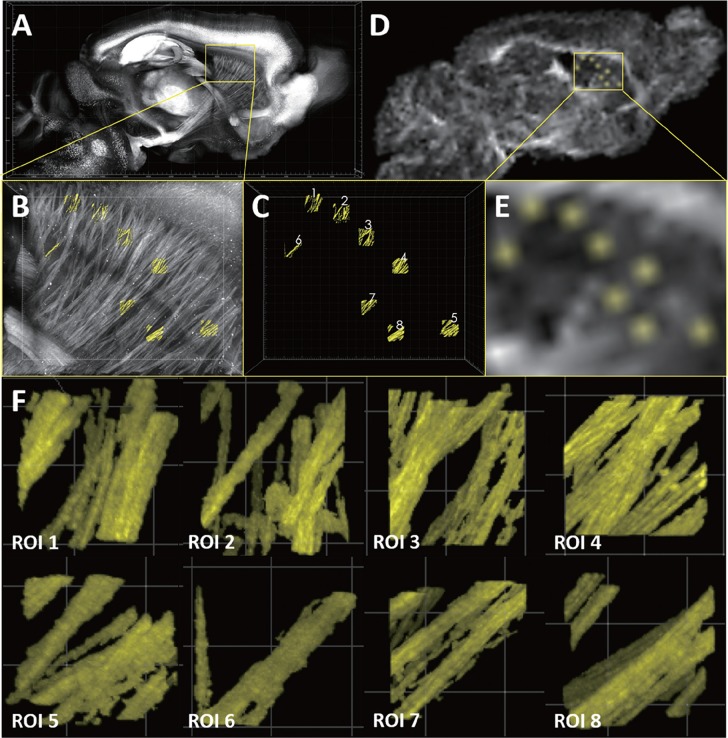
Two-photon imagings of cleared Thy-1 yellow fluorescent protein mouse brain (**A**, **B**, **C**, **F**) and fractional anisotropy (FA) map of the same mouse brain (**D**, **E**) (caudate nucleus and putamen). (**A**) Sagittal image 1 mm to the left of the midline acquired by two-photon microscopy. (**B**) Magnification of (**A**) centered on the caudate nucleus and putamen. The regions of interest (ROIs; yellow) were set randomly within the medial caudate putamen. (**C**) Neurites within the ROIs are shown in yellow. (**D**) FA map of the same cross-section as (**A**). (**E**) Magnification of (**D**). ROIs were set in the same regions as those shown in (**B**). (**F**) Histological details in selected ROIs. In all the ROIs there were numerous nerve fiber crossings.

The ROIs for measurement were selected in the medial lemniscus, where white matter fibers are more or less regularly aligned, and the caudate nucleus and putamen, which contain numerous crossing fibers. Eight ROIs each were set at random in the medial lemniscus and caudate nucleus and putamen in two-photon microscope image data (Figs. 1, 2). Each ROI measured 150 μm × 150 μm × 150 μm. Histological neurite density was calculated using Imaris Interactive Microscopy Image Analysis software (Bitplane AG, Zurich, Switzerland) with a threshold based surface reconstruction. Dark soma regions were discarded from the data. The threshold was slightly adjusted according to the microscopic image intensity (manual threshold over 2000 in grey value, number of voxels above 1000). The volume fraction was assumed to reflect the neurite density in each selected ROI. We manually identified the sagittal section on the FA map that corresponded to each sagittal image obtained by two-photon microscopy. White matter fibers with easily recognizable structures such as the corpus callosum, anterior commissure, and medial lemniscus were used in identifying the corresponding sagittal section on the FA map.

Once the corresponding sagittal sections had been identified, ROIs were created on the FA map, with care taken to confirm visually that the ROIs selected in the medial lemniscus and in the caudate nucleus and putamen were in the same anatomical positions as observed on the two-photon microscopy images. The size of the ROIs selected on the FA map was the same as that of the ROIs selected by two-photon microscopy (150 μm × 150 μm × 150 μm), and the FA and MD were measured (Figs. 1, 2, Table 1). The correlations between FA and MD values and neurite densities obtained from two-photon imaging data were then analyzed by calculating the Pearson correlation coefficients. The correlation was regarded as “very weakly positive,” “weakly positive,” “moderately positive,” “strongly positive,” and “very strongly positive” when *r* ranged from 0.00 to 0.20, above 0.20 to 0.40, above 0.40 to 0.60, above 0.60 to 0.80, and above 0.80 to 1.0, respectively.

## Results

**Fig. 3.**
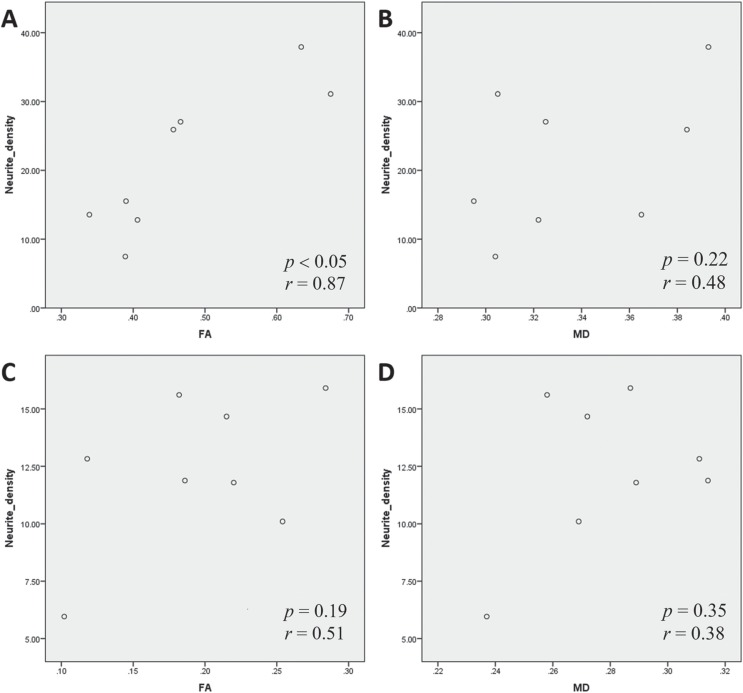
Comparison of neurite density measured by two-photon imaging and diffusion tensor parameters. (**A**) In the medial lemniscus, there was a very strong positive correlation between neurite density and FA (*P* < 0.05, *r* = 0.87). (**B**) In the medial lemniscus, there was a moderately strong positive correlation between neurite density and MD (*P* = 0.22, *r* = 0.48). (**C**) In the caudate nucleus and putamen there was a moderately positive correlation between neurite density and FA (*P* = 0.19, *r* = 0.51). (**D**) In the caudate nucleus and putamen there was a weakly positive correlation between neurite density and MD (*P* = 0.35, *r* = 0.38). FA, fractional anisotropy; MD, mean diffusivity.

The only significant correlation observed was between FA and neurite density in the medial lemniscus (Fig. 3A). There was no significant correlation between neurite density and either the MD in the medial lemniscus or the FA or MD in the caudate nucleus and putamen. In the medial lemniscus, there was a very strong positive relation between FA and neurite density (*r* = 0.87) (Fig. 3A), and a moderately positive relation between MD and neurite density (*r* = 0.48) (Fig. 3B). In the caudate nucleus and putamen there was a moderately positive relation between FA and neurite density (*r* = 0.51) and a weakly positive relation between MD and neurite density (*r* = 0.38) (Fig. 3C, D).

## Discussion

We found a very strong positive relation between FA (a commonly used DTI parameter) and neurite density in an area where nerve fiber orientations are unidirectionally aligned (the medial lemniscus). Mac Donald et al*.*^9^ also reported a significant correlation between FA and neurite density in ROIs located within a unidirectional axonal bundle, consistent with the present results. However, to our knowledge, our study is the first to verify this finding on the basis of 3D histological images obtained by brain clearing and two-photon microscopy, providing new pathological underpinnings for DTI. As expected, no significant correlation was observed between an FA and a neurite density in a region that contains crossing fibers (the caudate nucleus and putamen), and the relationship was only moderately positive. Traditional DTI is of limited value for identifying structures in regions that contain crossing fibers.^10^ If a voxel contains crossing fibers, the shape of the diffusion tensor will become more planar; consequently, the diffusion tensor parameters in regions that contain crossing fibers assume values lower than the actual neurite densities.^11,12^ Our results also suggest that the correlation between FA and neurite density in the caudate nucleus and putamen, which contain crossing fibers, was only moderately positive because FA values decreased relative to the actual neurite density as the proportion of crossing fibers increased. The correlation coefficients for MD were moderately to weakly positive in the medial lemniscus as well as the caudate nucleus and putamen. This is presumably because MD is a directionally an averaged measure of water diffusion^13^ and does not accurately reflect orientation-dependent water mobility in tissues such as white matter where the fiber orientation is strongly anisotropic. Consideration of other diffusion tensor parameters, including axial diffusivity and radial diffusivity, will likely be necessary in the future.

The brain clearing technique used here may also be useful for future studies in verifying novel diffusion MRI parameters such as those provided by diffusional kurtosis imaging^14^ and neurite orientation dispersion and density imaging.^15^ Limitations of our study include the facts that ROIs were set manually and that only a single sample was used. Future studies will need to verify the results by setting ROIs automatically through registration of microscopy images with MRI data and by using a larger number of samples. Furthermore, as a general limitation of research using brain clearing, the possibility that this treatment may have minute effects on brain structure must be considered. Because samples that have undergone brain clearing exhibit slightly large expansion, caution is required in evaluating microscopic cellular morphology. The fact that brain-clearing reagents also cause the loss or partial degeneration of cellular proteins must also be taken into consideration.

## Conclusion

We could verify the estimates of diffusion tensor parameters from 3D data obtained by brain clearing and two-photon microscopy. This verification may provide new histological underpinnings for DTI.

## Figures and Tables

**Table 1.** Diffusion tensor parameters and neurite densities of each ROI

Medial lemniscus	FA	MD	Neurite density (%)
ROI 1	0.456	0.384	25.93
ROI 2	0.466	0.325	27.05
ROI 3	0.339	0.365	13.57
ROI 4	0.634	0.393	37.93
ROI 5	0.390	0.295	15.53
ROI 6	0.675	0.305	31.11
ROI 7	0.389	0.304	7.47
ROI 8	0.406	0.322	12.80

Caudate nucleus and putamen			

ROI 1	0.215	0.272	14.67
ROI 2	0.182	0.258	15.61
ROI 3	0.118	0.311	12.83
ROI 4	0.284	0.287	15.91
ROI 5	0.186	0.314	11.88
ROI 6	0.102	0.237	5.96
ROI 7	0.254	0.269	10.10
ROI 8	0.220	0.289	11.79

Neurite density was measured by two-photon microscopy in a cleared mouse brain. FA, fractional anisotropy (dimensionless); MD, mean diffusivity (10 × −3 mm^2^/s); ROI, region of interest.
